# Potential Use of Melamine Phytate as a Flame-Retardant Additive in Chicken Feather-Containing Thermoplastic Polyurethane Biocomposites

**DOI:** 10.1021/acsomega.3c01754

**Published:** 2023-07-04

**Authors:** Aysenur Mutlu, Aysegul Erdem, Mehmet Dogan

**Affiliations:** † Department of Textile, Apparel and Leather Van Vocational School of Higher Education, Yuzuncu Yıl University, 65080 Van, Turkey; ‡ Department of Textile Engineering, Erciyes University, 38039 Kayseri, Turkey

## Abstract

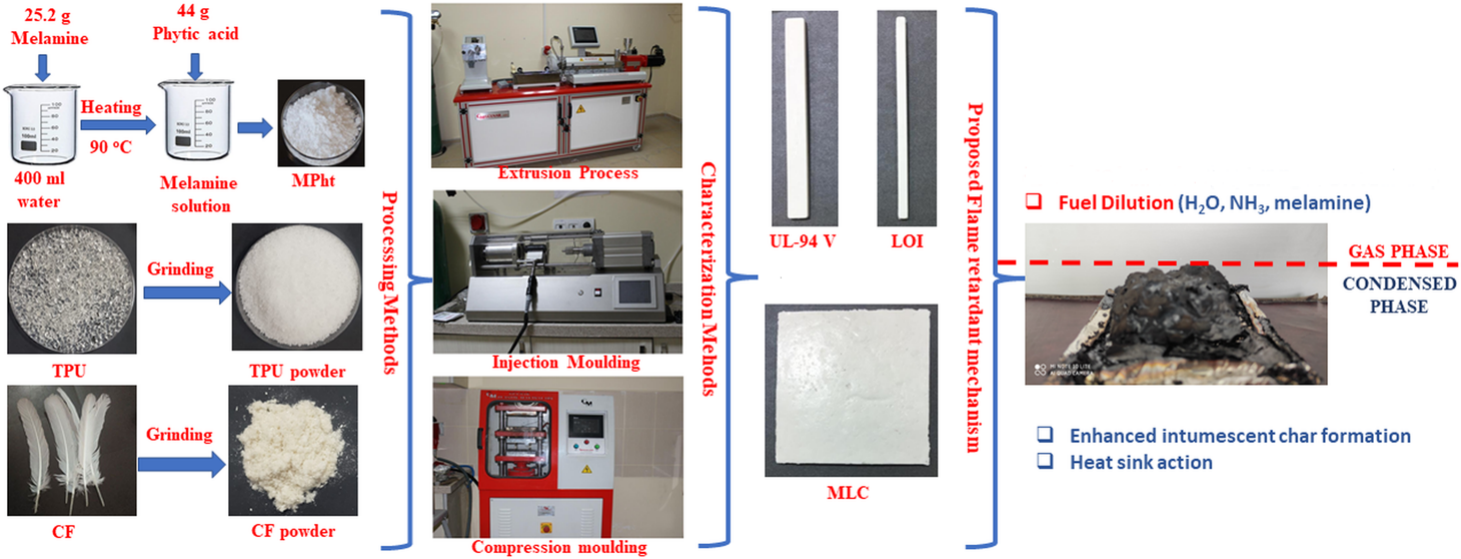

Using waste materials such as chicken feathers (CF) and biobased flame-retardant additives including melamine phytate (MPht) has become an effective approach for environmentally friendly and sustainable production in recent years. This study explores the flame retardant effectiveness of MPht in thermoplastic polyurethane (TPU)-based biocomposites containing CF. The characterizations of the composites are performed through thermal gravimetric analysis (TGA), limiting oxygen index (LOI), vertical UL-94 (UL-94 V), and mass loss calorimetry (MLC) tests. According to the test results, the highest UL-94 V rating of V0, a LOI value of 29.4%, and the lowest peak heat release rate (pHRR) (110 Kw/m^2^) and total heat evolved (THE) (39 MJ/m^2^) values are obtained with the use of 20 wt % MPht. It is demonstrated that MPht acts as an effective flame-retardant filler through the formation of intumescent char in the condensed phase and flame dilution in the gas phase.

## 1 Introduction

Thermoplastic polyurethane (TPU), a multiphase block copolymer composed of hard and soft segments, can be synthesized from polyols and diisocyanates derived from petrochemical and/or renewable resources. Because of the variety of available monomers, there are many different grades of TPU with varying properties. The outstanding properties of flexibility, convenient mass production, recyclable properties, and good abrasion resistance can give rise to finding use in numerous industrial sectors of medical, automobile, construction, electrical, and electronic. However, some drawbacks of TPU including poor thermal stability, low mechanical strength, and high flammability restrict its wider application. To address these issues, polymer blend technology and various types of fillers are used with TPU.^1,2^

About 40 million ton of feather-based waste materials is produced in a year. In recent years, finding unique and added value applications for them has attracted careful attention. Feather-based fibers are used in numerous applications including nonwovens, biochar, biodiesel, bioplastics, and composites owing to producing novel sustainable and ecofriendly materials with increasing environmental consciousness.^1,3,4^ In the development of biocomposites, feather fibers, such as chicken feather (CF), have been utilized and referenced in previous detailed review articles.^4−9^ Specifically, the incorporation of CF has been shown to enhance the mechanical properties of TPU in the literature.^1,10−13^

CF mainly consist of keratin (90%), water (1.8%), and oil (1.3%). Keratins, which are built from amino acids, especially cystine (∼10 wt % total amino acids), contain heteroatoms including nitrogen, oxygen, and sulfur. They leave a remarkable amount of char during the combustion.^14^ Accordingly, CF and keratin derived from CF have been utilized in the production of flame-retardant textiles and composites.^15,16^ In the literature, the flame retardant properties of CF-containing polypropylene-,^17−20^ TPU-,^21,22^ and polyester resin-based^23^ composites were improved using commercial flame retardants.

Several different additives including biomolecules were used to enhance the flame retardant properties of TPU-based composites.^24,25^ Biomolecules including phytic acid derivatives have been considered environmentally friendly flame retardant fillers in various polymeric materials.^15,16,26,27^ Phytic acid was used to modify boron nitride and silicon nitride, and the resulting modified compounds were used as flame-retardant additives in TPU.^28−30^ Phytic acid has the ability to form complexes with organic cations including melamine via coordinative bonds and supramolecular interactions.^31,32^ Melamine is classified in nitrogen-containing flame-retardant additives and is considered environmentally friendly due to the low evolution of smoke and low toxicity in the event of fire with the release of carbon dioxide, ammonia, and water.^33^ The flame retardant effect of melamine phytate (MPht) was investigated in polypropylene^34−38^ and poly(lactic acid).^39^

In the current study, MPht is selected as a biobased additive to produce sustainable and ecofriendly TPU-based biocomposites with an enhanced flame retardant character. The thermal and flame retardant performances of the biocomposites are analyzed using thermogravimetric analysis (TGA), limiting oxygen index (LOI), vertical UL 94 (UL-94V), and mass loss calorimetry (MLC) tests.

## 2 Experimental Studies

### 2.1 Materials

Polyether-based TPU with the commercial name PEARLTHANE CLEAR 15N85-CF was purchased from Brenntag (Istanbul, Turkey). It has a Shore A hardness of 86 and a density of 1.11 g/cm^3^. CF was supplied from local sources located in Bursa, Turkey. Phytic acid was purchased from Sigma Aldrich as a 50 wt % aqueous solution. Melamine with the trade name Melafine was purchased from DSM (Geleen, the Netherlands).

### 2.2 Synthesis of Melamine Phytate

MPht was synthesized using the starting materials melamine (25.2 g, 0.2 mol) and 50 wt % phytic acid solution (44 g, 0.033 mol). 0.2 mol melamine was dissolved in 400 mL of distilled water and heated up to 90 °C. Phytic acid solution was added to the heated solution and stirred for 30 min. The precipitated off-white MPht crystals were washed two times with distilled water and dried in an oven at 80 °C for 24 h. The reaction yield was about 60%. The characterization of MPht was performed using attenuated total reflection Fourier transform infrared spectroscopy (ATR-FTIR) and TGA analysis.

### 2.3 Production of the Composites

**Figure 1:**
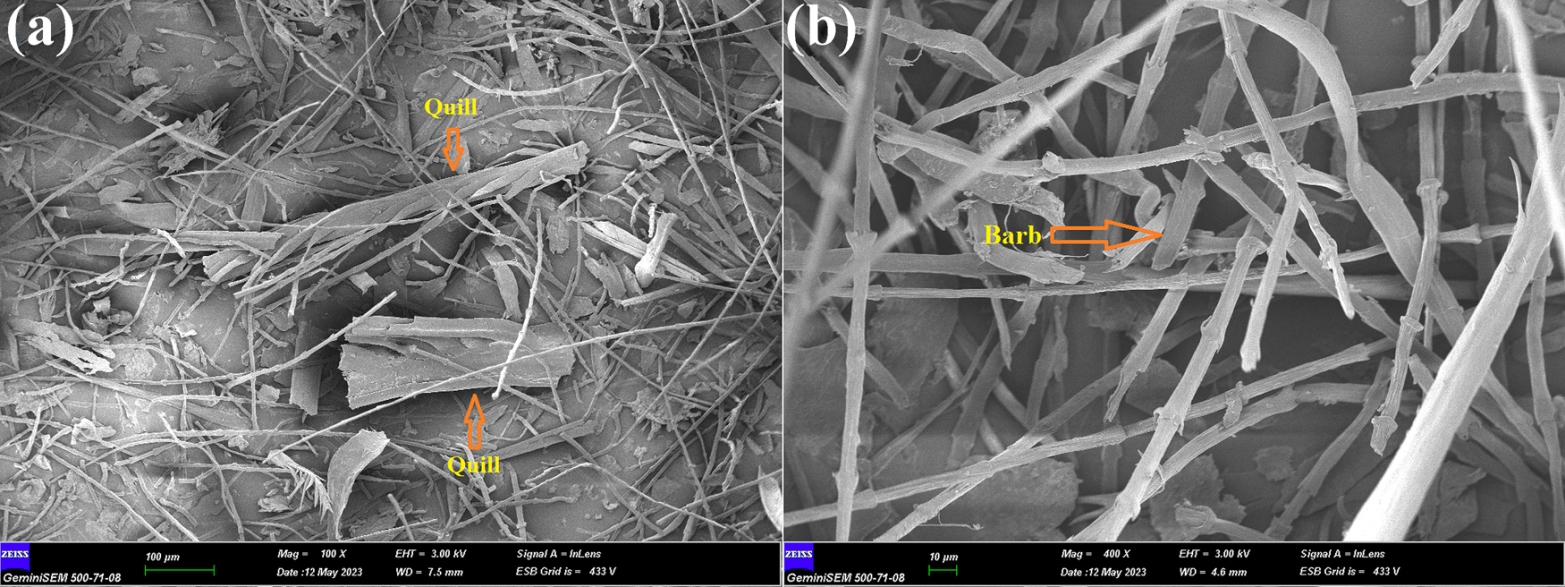
SEM images of ground CF with magnifications of (a) ×100 and (b) ×400.

CF were washed with hot soapy water (60 °C) two times before the grinding process to remove the oil and other unwanted stains. After the washing process, CF was dried at 80 °C for 16 h. CF with quills were ground with blade grinding (FRITSCH PULVERISETTE 19, Germany). After the grinding process, CF in the powder form were used in the composite production. The SEM images of ground CF with magnifications of ×100 and ×400 are shown in Figure 1a,b, respectively. As seen in Figure 1, barbs sustain their fiber form and quills were ground into small fragments. CF and MPht were dried in an oven at 80 °C for 24h prior to the compounding process. The extrusion process was performed in a twin screw extruder (GULNAR MAKINA, Istanbul, Turkey) at 100 rpm with the temperature profile of 50, 165, 170, 175, 170, and 165 °C from the hopper to the die. The samples for LOI, UL-94 V, and tensile tests were molded using a laboratory-scale injection-molding machine (DSM Xplore 12 mL Micro-Injection Molder, Netherlands). The barrel and mold temperatures and the injection pressure were constant at 190 °C, 30 °C, and 8 Bar, respectively. The samples for the MLC test were produced using a laboratory-scale hot-press (GULNAR MAKINA, Istanbul, Turkey) for 3 min at 160 °C. The flame retardant performance of MPht was examined in a constant CF loading of 20 wt %. MPht was used in three different concentrations of 5, 10, and 20 wt %. For sample coding, the abbreviations TPU, CF, and MPht were used for thermoplastic polyurethane, chicken feather, and melamine phytate, respectively. The code TPU/CF/10 MPht indicates the sample containing 20 wt % CF and 10 wt % MPht.

### 2.4 Characterization Methods

ATR-FTIR analysis was performed for melamine, phytic acid, MPht, heated MPht at 800 °C, and the char residues remaining after the MLC test at an optical resolution of 4 cm^–1^ with 32 scans. TGA tests were carried out for each component (TPU, CF, and MPht) and the composites using a Hitachi-High Tech STA-7300 instrument with a heating rate of 10 °C/min from room temperature to 800 °C under a nitrogen flow of 50 mL/min. LOI values were examined using a Fire Testing Technology (FTT) Limiting Oxygen Index Analyzer instrument on the test bars of 130 × 6.5 × 3.2 mm^3^ in size, according to the standard oxygen index test ASTM D2863. The UL 94 V test was performed on the test bars of 130 × 13 × 3.2 mm^3^ according to ASTM D3801. The MLC test was carried out using Mass Loss Cone with a thermopile attachment (FTT, U.K.) under a heat flux of 35 kW/m^2^ according to the ISO 13927 standard. Square specimens with a dimension of 100 × 100 × 3 mm^3^ were used. Tensile properties of the composites were analyzed at room temperature using a Devotrans GP/R testing machine equipped with a 5 kN load cell, following the ASTM D 638 standard. The tests were carried out on dog bone-shaped samples (7.4 × 2.1 × 80 mm^3^) at a crosshead speed of 50 mm/min. The tensile strength and percentage elongation at break values were recorded, and the results were averaged over five samples with standard deviations. The microstructures of ground CF and the residual chars remaining after the MLC test, and the tensile fracture surfaces of the composites were examined with SEM (FEI Quanta 400F). The samples were coated with gold with a sputter coater to achieve the conductivity.

## 3 Results and Discussion

### 3.1 Characterization of Melamine Phytate

**Figure 2:**
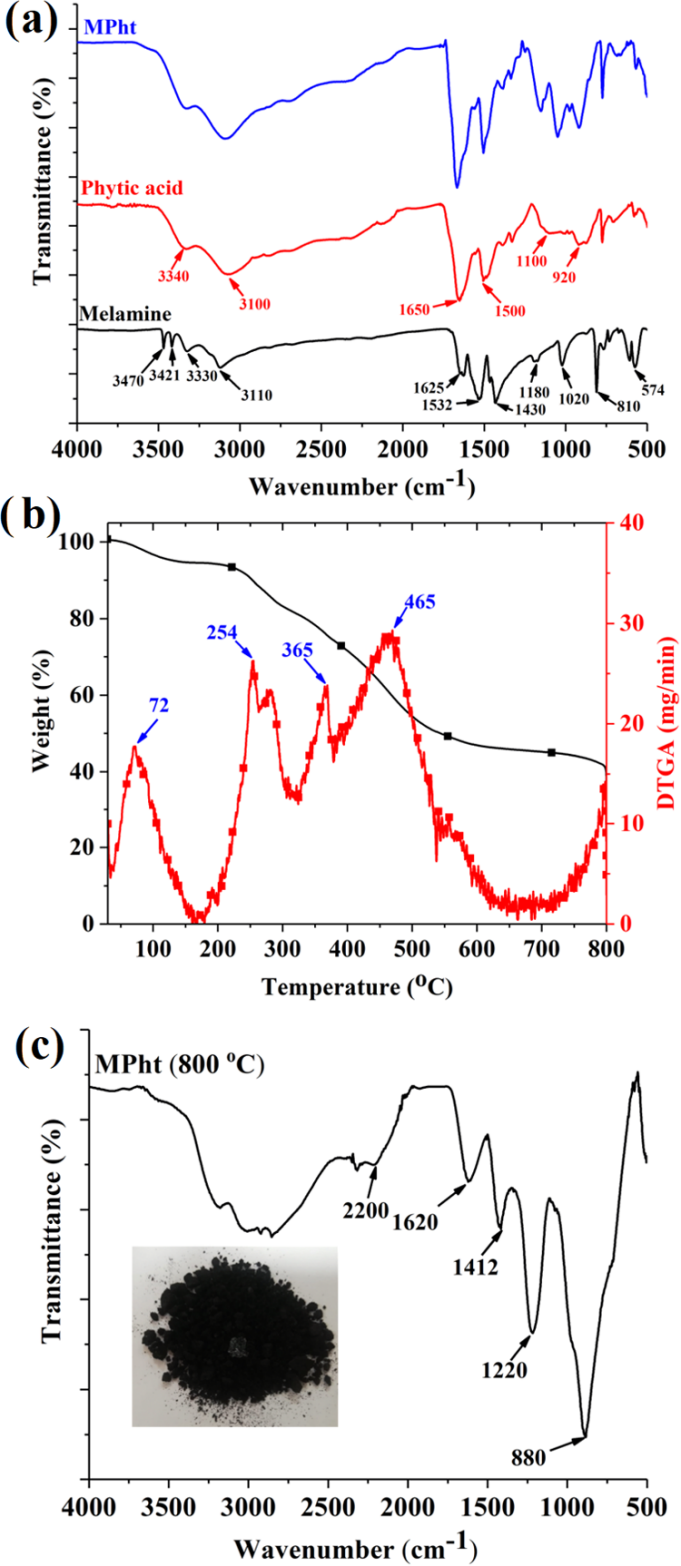
(a) FTIR spectra of melamine, phytic acid, and MPht, (b) TGA and DTGA curves of MPht, and (c) FTIR spectra and digital photograph of MPht heated at 800 °C.

**Figure 3:**
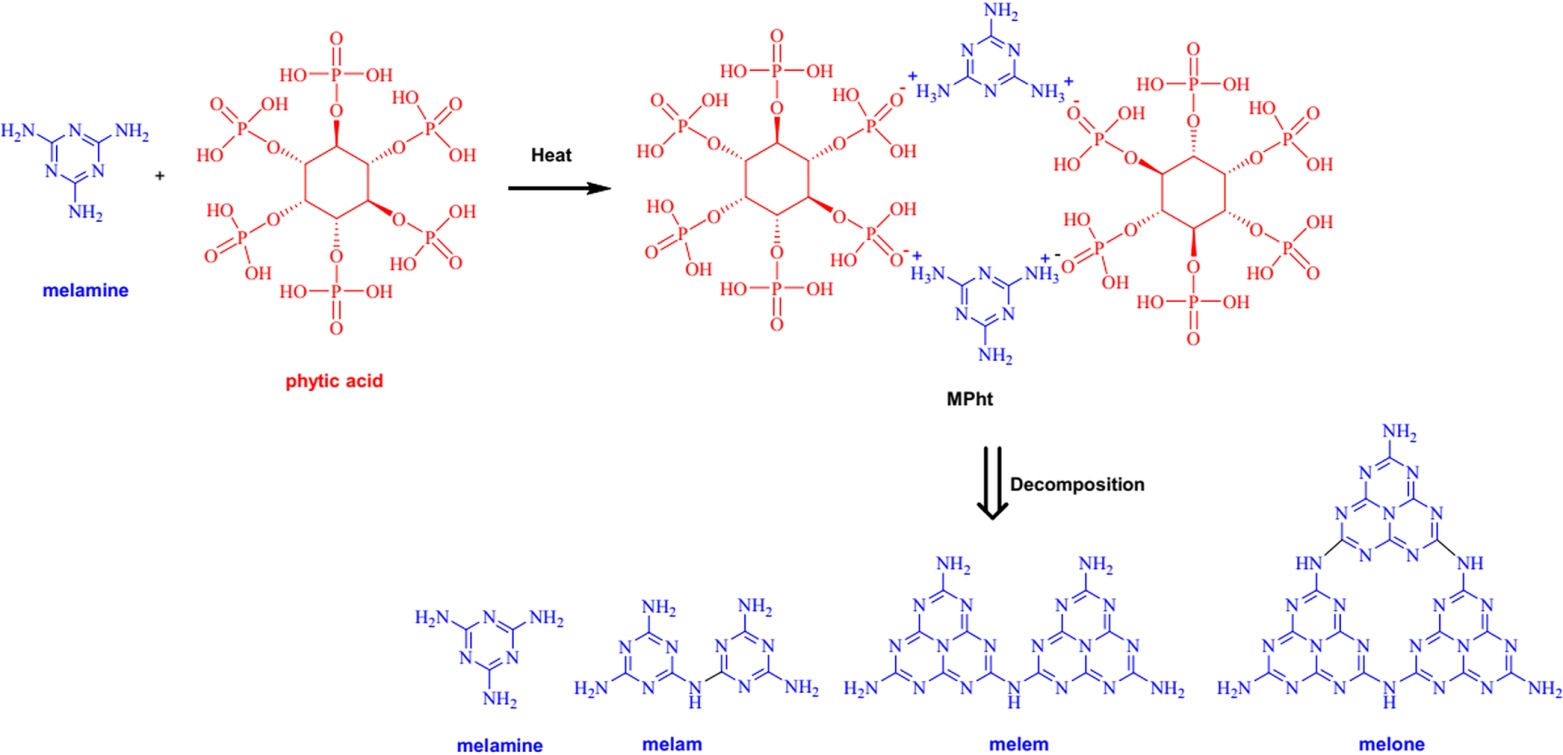
Molecular structure of MPht and the thermally stable condensates after decomposition.

ATR-FTIR analysis was used to confirm the synthesis of MPht, and the spectra of melamine, phytic acid, and MPht were compared. The related spectra are given in Figure 2a. As seen from Figure 2a, melamine displays characteristic peaks at 3470, 3421, 3330, 3110, 1625, 1532, 1430, 1180, 1020, 810, and 574 cm^–1^. The peaks seen at 3470, 3421, and 3110 cm^–1^ stem from the typical symmetric stretching vibrations of the NH_2_ group. The peak observed at 3330 cm^–1^ arises from the asymmetric stretching vibrations of the NH_2_ group. The peak seen at 1625 cm^–1^ is attributed to the NH_2_ group deformation. The other peaks observed at 1532, 1430, 1180, 1020, 810, and 574 cm^–1^ are attributed to the stretching and bending vibrations occurring in the triazine ring.^37,40^ Phytic acid has characteristic peaks appearing at 3340, 3100, 1650, 1500, and 920 cm^–1^. The peak seen at 3340 cm^–1^ is attributed to the stretching vibration of the −OH group. The peaks seen at 3100 and 1500 cm^–1^ are caused by C–H stretching and bending vibrations of the CH_3_ group, respectively. The peaks observed at 1650, 1100, and 920 cm^–1^ are caused by the stretching vibrations of O–P–O, C–O–P, and P=O groups, respectively.^41,42^ MPht exhibits the characteristic peaks of both melamine and phytic acid, which mask each other. The most notable difference is seen as the disappearance of the peaks seen at 3470 and 3421 cm^–1^ owing to the protonation of the NH_2_ group (NH_3_^+^) during the complex formation between melamine and phytic acid. The ATR-FTIR result is consistent with the literature.^34,37,39^ The molecular structure of MPht is shown in Figure 3.

TGA and DTGA graphs of MPht are shown in Figure 2b, and the related data are given in Table 1. As seen in Figure 2b, MPht decomposes in four successive steps. In the first step (72 °C), evolution of the physically absorbed water occurs. Melamine salts decompose with two competing pathways of thermal dissociation and decomposition of each component via condensation reactions depending upon the parent acid type.^43^ The phosphorus-containing acids favor the condensation of melamine as in the case of phytic acid.^43,44^ The second degradation step takes place at 254 °C with a shoulder at 280 °C. In the second step, the sublimation and condensation of melamine and the dehydration and carbonization of phytic acid occur simultaneously. In the second step, a thermally stable melam structure up to 350 °C is formed. Melam further undergoes successive condensation reactions in the third and fourth steps with the formation of melem (stable up to 450 °C) and melon (stable up to 600 °C) with the release of NH_3_^45^ Finally, a thermally stable cyameluric ring formed. The structures of thermally stable condensates are shown in Figure 3. In the fourth step, pyrophosphate- and polyphosphate-based compounds are also formed with the degradation of phytic acid as well.^37,46,47^ MPht leaves 37.4 wt % nitrogen and phosphorus-containing residue at 800 °C. In order to indicate the residue structure, MPht is heated in an oven at 800 °C for 30 min. After the treatment, the color of MPht turns black (Figure 2c). ATR-FTIR spectra of the residue are given in Figure 2c. The residue has characteristics peaks at 2200, 1620, 1412, 1220, and 880 cm^–1^. The peak seen at 2200 cm^–1^ is attributed to the cyanate group. As stated in the literature, the cyameluric ring has characteristic peaks at 1620, 1412, and 800 cm^–1^. The peaks seen at 1220 and 880 cm^–1^ stem from the P=O and P–O–P groups in the pyrophosphate and polyphosphate structures, respectively.^43,44^

**Table 1:** TGA Data of the Fillers, Polymer, and Composites

sample	*T*_5%_ (°C)^a^	*T*_max1_ (°C)^b^	*T*_max2_ (°C)^b^	*T*_max3_ (°C)^b^	*T*_max4_ (°C)^b^	residue yield calc. (%)^c^	residue yield exp. (%)^d^
MPht	95	72	254	365	465		36.8
CF	88	68	311				21.7
TPU	318	402					2.3
TPU/CF	279	322	400				6.6
TPU/CF/5 MPht	263	313	380			7.9	20.5
TPU/CF/10 MPht	258	307	365			9.7	22.1
TPU/CF/20 MPht	262	304	359			13.1	29.1

a Temperature at 5% weight loss.

b The maximum degradation rate temperatures.

c Char Yield at 800 °C (calculated).

d Char Yield at 800 °C (experimental).

### 3.2 Thermal Decomposition of the Composites

**Figure 4:**
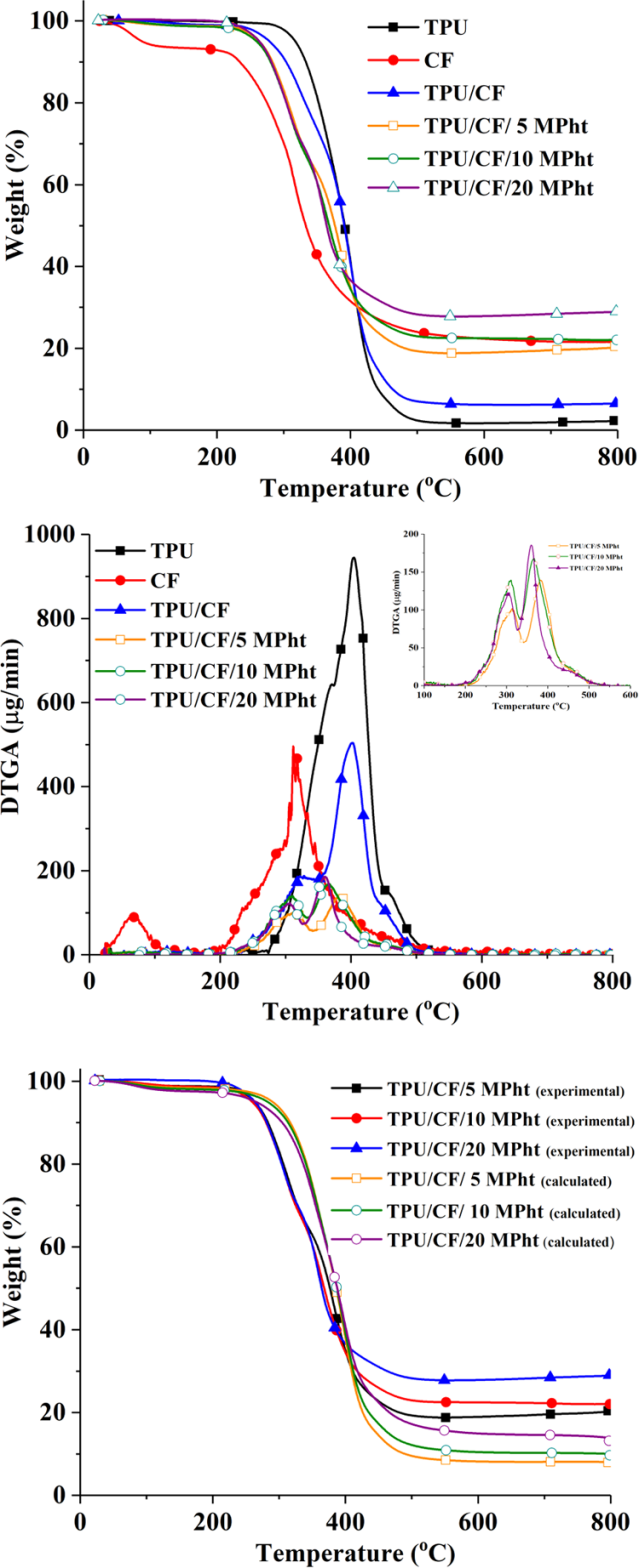
Experimental TGA and DTGA graphs and the calculated TGA graphs of the composites.

Thermal decomposition characteristics of CF, TPU, and the composites are examined via TGA analysis under a nitrogen atmosphere. The related data are stated in Table 1. Figure 4 displays the TGA and DTGA graphs obtained experimentally as well as the calculated TGA curves. As seen from Figure 4, CF decomposes in two steps with the maximum decomposition rates at 68 and 311 °C leaving 21.7 wt % residue based on heterocyclic amines and aromatic structures via crosslinking, cyclization, and aromatization reactions.^48,49^ The first step is attributed to the removal of the physically absorbed water, while the second step taking place with successive overlapped degradation steps of keratin just starts from 200 °C and ends at about 500 °C via cleavage of the disulfide and peptide bonds and the aforementioned char-forming reactions. In the detailed analysis performed by Senoz et al.,^48^ and Brebu and Spiridon^49^ showed that various aqueous and volatile combustible and noncombustible (water, ammonia, and carbon dioxide) decomposition products were formed during the decomposition.

Neat TPU decomposes in a single step, leaving 2.3 wt % carbonaceous residue. With the addition of CF into TPU, the composite degrades in two steps at 322 (shown as a shoulder) and 400 °C. The first and second steps stem mainly from the degradation of CF and TPU, respectively. The addition of CF reduces the initial thermal stability (*T*_5%_) of TPU at about 40 °C owing to the lower thermal stability of CF. The residue yield increases from 2.2 to 6.6 wt % due to the solid decomposition products of CF.

MPht-containing composites also undergo two-step degradation. The addition of MPht further reduces the *T*_5%_ value of the composites. The maximum degradation temperatures (*T*_max1_ and *T*_max2_) decrease as the added amount of MPht increases due to the formation of phytic acid during the decomposition of MPht. It is concluded that phytic acid accelerates the degradation of CF and TPU. The addition of MPht improves the residue yield as the added amount increases. The experimental residue yields are found to be higher than the calculated ones, indicating the presence of interactions among MPht, CF, and TPU. It is a well-known fact that phosphorus-containing additives promote the char formation in heteroatom-containing polymers including CF and TPU. Similar findings, reduction in thermal stability and enhancement in residue yield, are also observed in the literature with the use of phosphoric acid with CF,^17,19^ phytic acid with silk/wool blend,^50^ and phytic acid-modified boron nitrides in TPU.^28−30^

### 3.3 Mass Loss Calorimetry Studies

**Figure 5:**
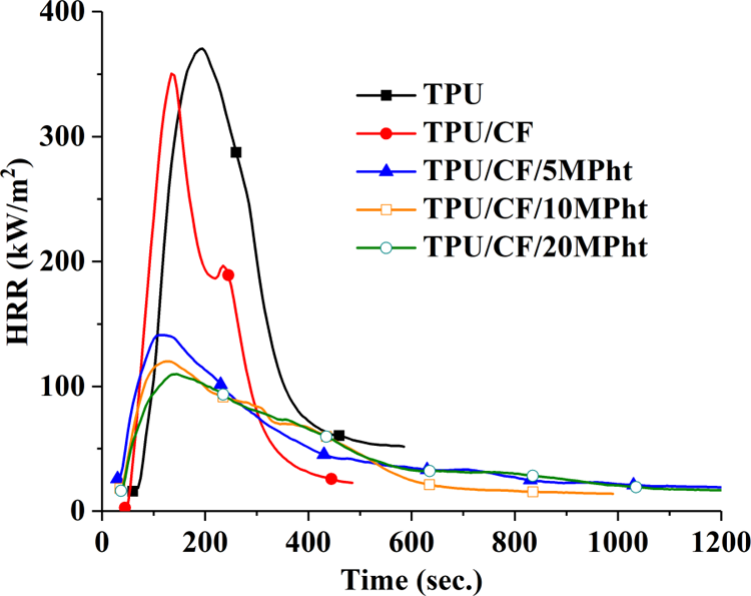
HRR curves of the composites.

**Figure 6:**
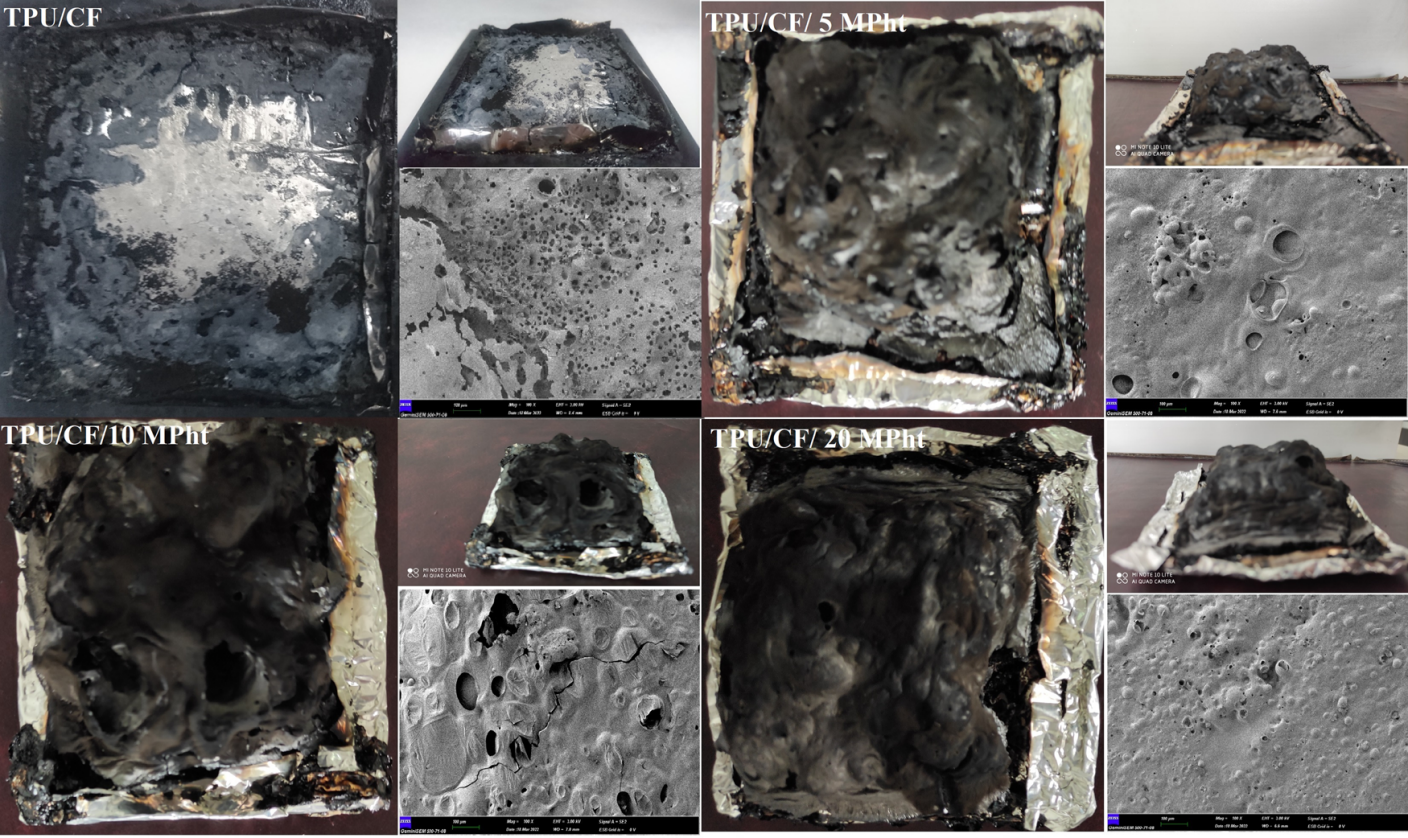
Photographs and SEM images (×100 magnification) of char residues.

MLC studies are considered a common approach to check and interpret the fire retardant performances of polymeric materials. The MLC test is conducted on all composites, and the average values are stated in Table 2. The performances of the composites are evaluated using time to ignition (TTI), peak heat release rate (pHRR), average HRR (avHRR), total heat evolved (THE) and THE/TML (total mass loss) ratio, and residue yield. HRR curves of the composites are shown in Figure 5. The digital photographs and SEM images (100× magnification) of the samples remaining after the MLC test are shown in Figure 6.

**Table 2:** MLC Data of the Composites

sample	TTI (s)	pHRR (kW/m^2^)	AvHRR (kW/m^2^)	THE (MJ/m^2^)	THE/TML (MJ/m^2^g)	residue (%)
TPU	63	371 ± 9	244 ± 8	68 ± 2	2.1	1.8
TPU/CF	48	350 ± 8	197 ± 7	54 ± 2	2.0	5.7
TPU/CF/5 MPht	31	141 ± 5	56 ± 4	47 ± 1	1.8	24.1
TPU/CF/10MPht	36	120 ± 5	60 ± 3	43 ± 2	1.7	27.6
TPU/CF/20 MPht	37	110 ± 6	52 ± 3	39 ± 2	1.6	32.8

As seen in Figure 5, TPU burns rapidly, giving a sharp HRR peak after ignition, and leaves 1.8% carbon-based residue. With the addition of CF, the HRR curve shifts left due to the reduction in the TTI value. The reduction in the TTI value arises from the lower thermal stability of CF with respect to TPU. Accordingly, the required amount of combustible volatile compounds for ignition reaches in a short time. A similar trend is observed with the use of protein-based fibers in polypropylene^19^ and epoxy resin.^51^ With the addition of CF, a little amount of char is formed and accumulated on the sides of aluminum foil as seen in Figure 6. Accordingly, the protective function of the char structure is not good, and no significant change is observed in the pHRR value. However, the THE value reduces about 14% due to the improved char formation. No meaningful change is observed with the addition of CF in the THE/TML value that gives information about the gas-phase action of additives.

**Figure 7:**
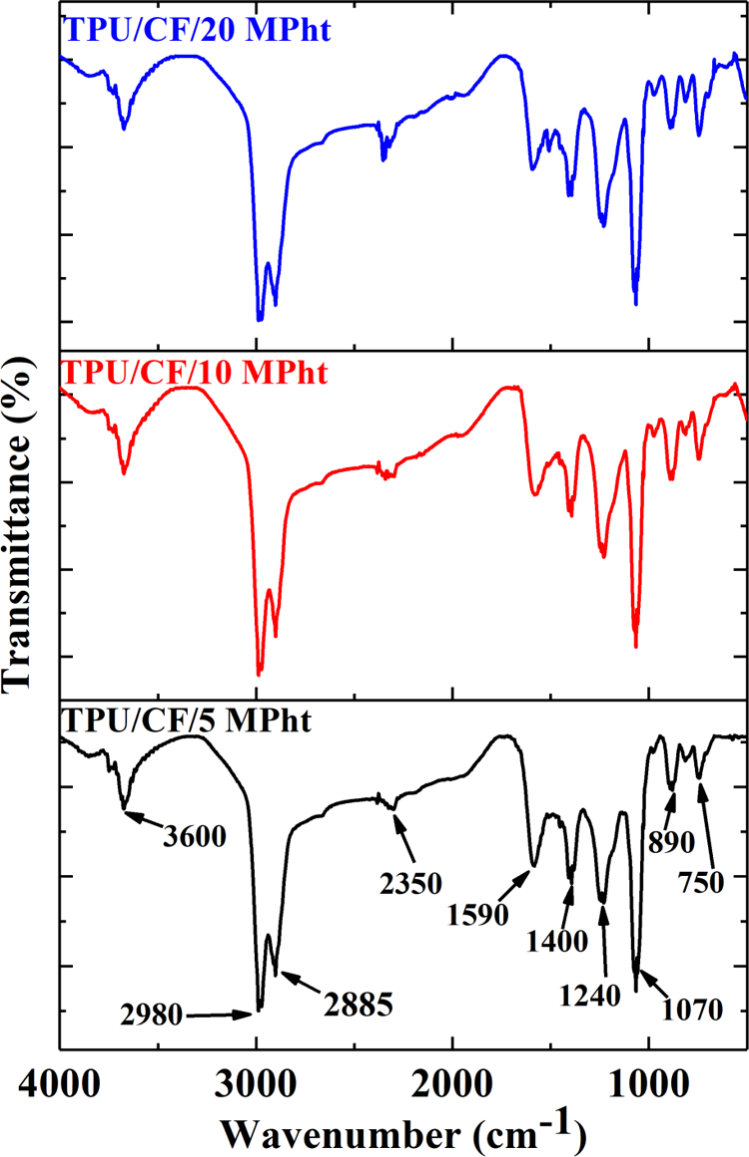
FTIR spectra of the residues.

With the addition of MPht, HRR curves become more plateau-like with prolonged combustion time. The addition of MPht causes reduction in TTI, pHRR, avHRR, THE, and THE/TML values. The lower TTI value with respect to TPU/CF arises from reduction in thermal stability as shown in TGA analysis in detail. The pHRR value reduces about 60, 66, and 69% with the addition of 5, 10, and 20 wt % MPht, respectively. The reduction in the pHRR value stems mainly from the decrease in the fuel source with improved char formation and the barrier effect of the compact char structure with a remarkable intumescent character, as seen in Figure 7. The THE value reduces steadily at about 13, 20, and 28% with increasing MPht amount. The reduction in the THE value is mainly attributed to the reduction in the fuel source. The reduction in the fuel source arises from three factors of the reduced TPU content with increasing MPht amount, enhanced char formation, and incomplete pyrolysis due to the barrier effect of the foamed char structure. In order to indicate char-forming interactions and incomplete pyrolysis, ATR-FTIR analysis is performed for the residues. The related spectra are shown in Figure 7. As seen from Figure 7, all residues have the same characteristic peaks. The characteristic peaks seen in heated MPht at 800 °C (see Figure 2) are also observed in the residues at 2350, 1590, 1400 1240, and 890 cm^–1^. It is concluded that a thermally stable cyameluric ring is also formed during the combustion under MLC test conditions. However, additional distinct sharp peaks are observed at 750, 1070, 2885, and 2980 cm^–1^ in the residue structures. The peaks observed at 1070 and 750 cm^–1^ are attributed to the stretching vibrations C–O and P–O groups in the P–O–C structure, which indicates char favoring interactions between MPht and TPU.^52^ The peaks seen at 2885 and 2980 cm^–1^ arise from stretching vibrations of the C–H group in aliphatic chain fragments due to the incomplete pyrolysis.^53,54^

The THE/TML value gradually reduces with increasing amount of MPht. The reduction of this value stems mainly from the flame dilution effect of MPht in the gas phase owing to the formation of noncombustible volatile compounds (H_2_O, NH_3_, and melamine) during the decomposition of MPht.

### 3.4 Flammability Properties

**Figure 8:**
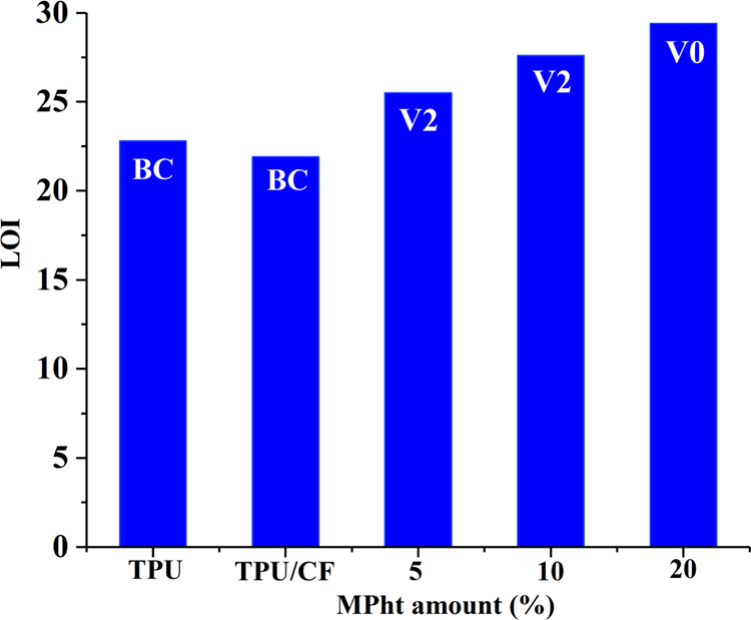
UL 94 V rating and LOI value of the composites.

The flammability characteristics of the composites are evaluated with LOI and UL-94 V tests. The related results are depicted in Figure 8. TPU burns to clamp (BC) in the UL-94 V test and has 22.8% LOI value. The addition of CF does not change the UL-94 V rating, and slight reduction in the LOI value (21.9%) is observed. With the addition of MPht, the LOI value steadily increases. The highest LOI value of 29.4% is observed with the addition of 20 wt % MPht. The V2 rating is achieved up to 10 wt % MPht addition. The highest UL-94 V rating of V0 is obtained with the addition of 20 wt % MPht. As a result of flammability tests, MPht is highly effective in TPU/CF composites. From detailed experimental studies, it is observed that the MPht exerts its flame retardant action predominantly in the condensed phase and slightly in the gas phase through the different mechanisms of heat sink action (endothermic decomposition), fuel dilution (H_2_O, NH_3_, and melamine), improved char formation, and protective residue formation with intumescent character.

### 3.5 Tensile Properties

**Figure 9:**
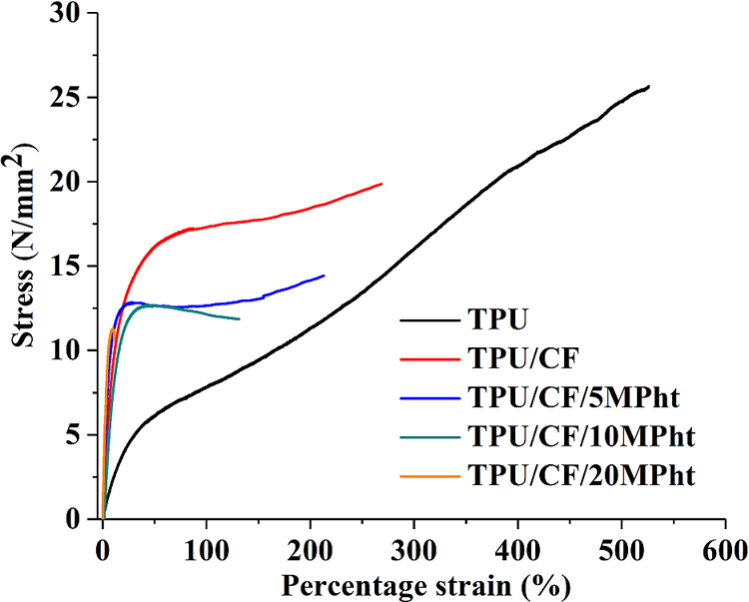
Stress–strain curves of the composites.

**Figure 10:**
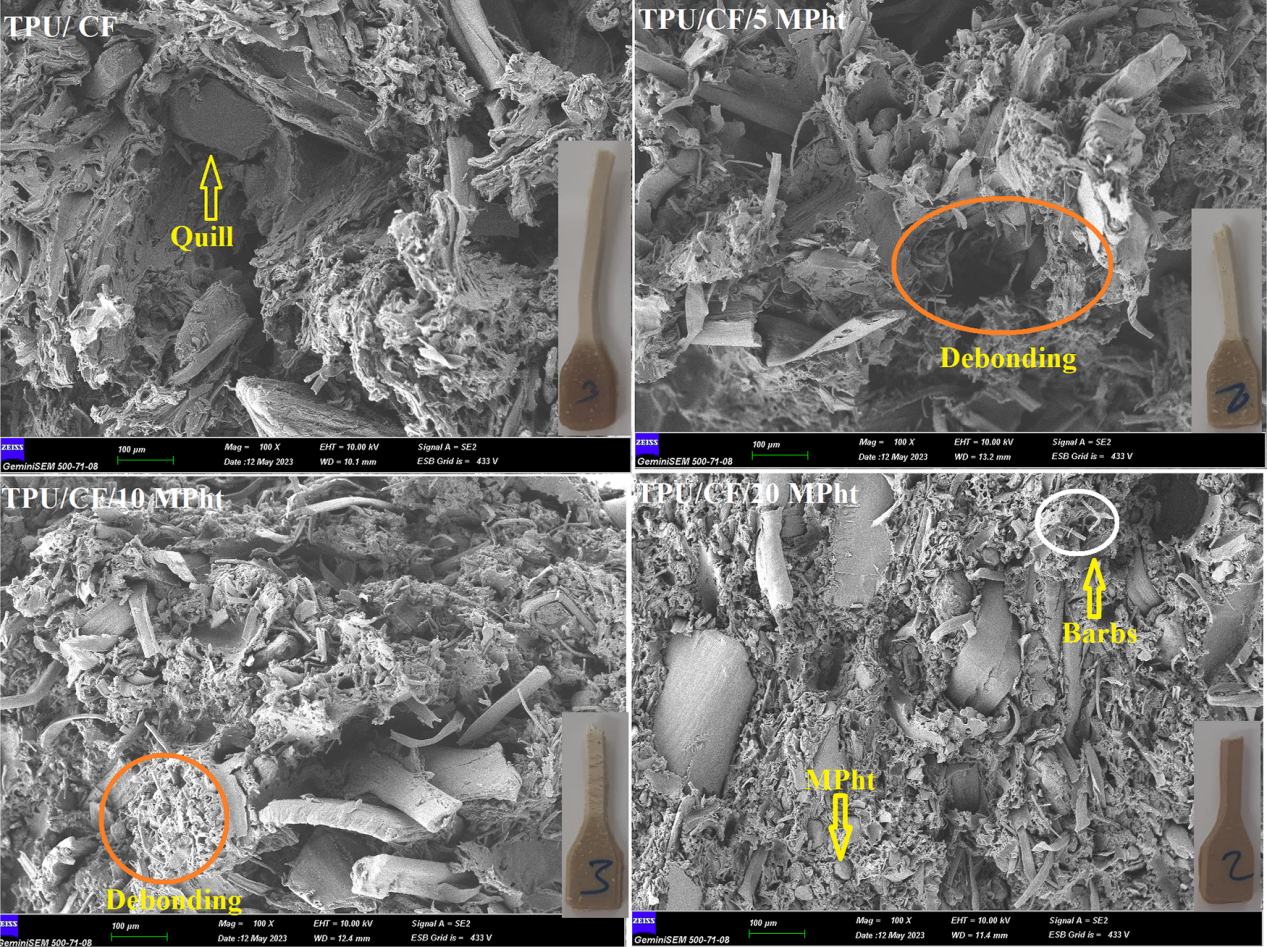
Photographs of tested tensile specimens and tensile-fractured surfaces of the composites with a magnification of ×100.

Tensile testing is carried out to understand the mechanical properties of the composites. The representative stress–strain curves of the composites are depicted in Figure 9. As seen in Figure 9, TPU has a tensile strength of 25.5 ± 1.5 N/mm^2^ and percentage strain of 525 ± 35 with strain hardening character. Reductions in tensile strength and percentage strain are observed with the addition of CF. However, the composite still has strain hardening character. Tensile strength and percentage strain are reduced at about 22 and 50%, respectively. With increasing amount of MPht, the gradual reductions in tensile strength and percentage strain are observed. It is thought that the formation and the growth of the pores around filler particles (barbs, large quill fragments, and MPht) give rise to premature failure of TPU. The strain hardening character diminishes when the added amount of MPht reaches 10 wt %. The composite containing 20 wt % MPht fails in a brittle manner. Tensile strength is reduced at about 23, 29, and 38% with respect to only CF-containing composites with the addition of 5, 10, and 20 wt % MPht, respectively. Tensile fracture surfaces of the composites give valuable information related with the observed trend. The photographs of the tested tensile specimens and the tensile-fractured surfaces of the composites with a magnification of ×100 are shown in Figure 10. As seen from Figure 10, TPU undergoes plastic deformation, and stress-induced whitening is observed up to 10 wt % MPht addition. Embedded and debonded barbs (seen as circular small holes) and quills (seen as large holes) are observed on the fracture surfaces. The large quill fragments and debonding due to the weak interfacial adhesion between CF and TPU cause reduction in tensile strength.

## 4 Conclusions

In the current study, the synthesized MPht was used as a biobased flame-retardant additive in CF-containing TPU composites. The effect of the MPht amount on the thermal and flame retardant characteristics of the composites was examined using TGA, LOI, UL 94V, and MLC tests. According to TGA analysis, the addition of CF and MPht reduced the thermal stability of TPU. MPht favored the char formation, and the residue yield increased as the added amount increased. According to the flammability test results, the LOI value steadily increased as the added amount of MPht increased. The V2 rating in the UL 94 V test was obtained in 5 and 10 wt % MPht-containing composites. The highest UL-94 V rating of V0 was achieved with the addition 20 wt % MPht. According to the MLC test results, the addition of MPht improved the fire retardant performance of the composite as the added amount increased with low pHRR, THE, and THE/TML values. In brief, MPht was highly effective in CF-containing TPU composites. It had a dual flame retardant effect observed in the condensed and gas phases via a heat sink, enhanced intumescent char formation, and flame dilution. According to tensile test results, the addition of CF and MPht gives rise to reduction in tensile strength and percentage strain.

## References

[ref1] Khalifa M.; Anandhan S.; Wuzella G.; Lammer H.; Mahendran A. R. Thermoplastic polyurethane composites reinforced with renewable and sustainable fillers–a review. Polym.-Plast. Technol. Mater. 2020, 59, 1751–1769. 10.1080/25740881.2020.1768544.

[ref2] Datta J.; Kasprzyk P. Thermoplastic polyurethanes derived from petrochemical or renewable resources: A comprehensive review. Polym. Eng. Sci. 2018, 58, E14–E35. 10.1002/pen.24633.

[ref3] Rogovina S. Z.; Prut E.; Berlin A. Composite materials based on synthetic polymers reinforced with natural fibers. Polym. Sci., Ser. A 2019, 61, 417–438. 10.1134/S0965545X19040084.

[ref4] Mrajji O.; El Wazna M.; El Bouari A.; Cherkaoui O. The Properties of Feather Fiber-Reinforced Polymer Composites: A Review. J. Nat. Fibers 2021, 19, 4868–4885. 10.1080/15440478.2020.1870633.

[ref5] Carrillo F.; Rahhali A.; Canavate J.; Colom X. Biocomposites using waste whole chicken feathers and thermoplastic matrices. J. Reinf. Plast. Compos. 2013, 32, 1419–1429. 10.1177/0731684413500546.

[ref6] Andrew J. J.; Dhakal H. Sustainable biobased composites for advanced applications: recent trends and future opportunities–A critical review. Compos., Part C: Open Access 2022, 7, 100220 10.1016/j.jcomc.2021.100220.

[ref7] Kurien R. A.; Biju A.; Raj K. A.; Chacko A.; Joseph B.; Koshy C. P. Chicken feather fiber reinforced composites for sustainable applications. Mater. Today: Proc. 2022, 58, 862–866. 10.1016/j.matpr.2021.10.400.

[ref8] Bansal G.; Jain Y.; Ahmed Y.; Kishore C.; Agarwal V. A comprehensive study on experimental analysis and development methods of chicken feather fiber reinforced bio composites. Mater. Today: Proc. 2021, 46, 10310–10314. 10.1016/j.matpr.2020.12.455.

[ref9] Khan A. A.; Parikh H.; Qureshi M. R. N. A Review on Chicken Feather Fiber (CFF) and its application in Composites. J. Nat. Fibers 2022, 19, 12565–12585. 10.1080/15440478.2022.2073495.

[ref10] Soykan U. Development of turkey feather fiber-filled thermoplastic polyurethane composites: Thermal, mechanical, water-uptake, and morphological characterizations. J. Compos. Mater. 2022, 56, 339–355. 10.1177/00219983211056137.

[ref11] Pourjavaheri F.; Jones O. A.; Czajka M.; Martinez-Pardo I.; Blanch E. W.; Shanks R. A. Design and characterization of sustainable bio-composites from waste chicken feather keratin and thermoplastic polyurethane. Polym. Compos. 2018, 39, E620–E632. 10.1002/pc.24794.

[ref12] Pourjavaheri F.; Jones O.; Mohaddes F.; Sherkat F.; Gupta A.; Shanks R. A. Green Plastics: Utilizing Chicken Feather Keratin in Thermoplastic Polyurethane Composites to Enhance Thermo-Mechanical Properties, Proceedings of the 74th Annual Technical Conference of the Society of Plastics Engineers, 2016; pp 1–8.

[ref13] Gokce O.; Kasap M.; Akpinar G.; Ozkoc G. Preparation, characterization, and in vitro evaluation of chicken feather fiber–thermoplastic polyurethane composites. J. Appl. Polym. Sci. 2017, 134, 45338 10.1002/app.45338.

[ref14] Tesfaye T.; Sithole B.; Ramjugernath D.; Chunilall V. Valorisation of chicken feathers: Characterisation of chemical properties. Waste Manage. 2017, 68, 626–635. 10.1016/j.wasman.2017.06.050. 28687152

[ref15] Basak S.; Ali S. W. Sustainable fire retardancy of textiles using bio-macromolecules. Polym. Degrad. Stab. 2016, 133, 47–64. 10.1016/j.polymdegradstab.2016.07.019.

[ref16] Villamil Watson D. A.; Schiraldi D. A. Biomolecules as flame retardant additives for polymers: A review. Polymers 2020, 12, 849 10.3390/polym12040849. 32272648 PMC7240707

[ref17] Jung D.; Bhattacharyya D. Keratinous fiber based intumescent flame retardant with controllable functional compound loading. ACS Sustainable Chem. Eng. 2018, 6, 13177–13184. 10.1021/acssuschemeng.8b02756.

[ref18] Jung D.; Kim N.; Bhattacharyya D. Use of Modified Chicken Feather to Enhance Flame Retardancy and Mechanical Properties of Polymeric Composites, AIP Conference Proceedings; AIP Publishing LLC, 2020 020028.

[ref19] Jung D.; Persi I.; Bhattacharyya D. Synergistic effects of feather fibers and phosphorus compound on chemically modified chicken feather/polypropylene composites. ACS Sustainable Chem. Eng. 2019, 7, 19072–19080. 10.1021/acssuschemeng.9b04894.

[ref20] Mishra A.; Jung D.; Kim N. K.; Bhattacharyya D. Influence of chicken feather fibre processing technique on mechanical and fire performances of flame-retardant polypropylene composites. Composites, Part A 2023, 165, 107338 10.1016/j.compositesa.2022.107338.

[ref21] Mutlu A.; Tayfun U.; Dogan M. Performance evaluation of melamine derivatives as flame retardant additive in chicken feather containing thermoplastic polyurethane biocomposites. J. Thermoplast. Compos. Mater. 2022, 08927057221133090 10.1177/08927057221133090.

[ref22] Mutlu A.; Dogan M. The effect of phosphorus based flame retardants on the thermal and fire retardant properties of chicken feather/thermoplastic polyurethane biocomposites. 2022.

[ref23] Kuru D.; Borazan A.A.; Guru M. Effect of chicken feather and boron compounds as filler on mechanical and flame retardancy properties of polymer composite materials. Waste Manage. Res. 2018, 36, 1029–1036. 10.1177/0734242X18804041. 30319051

[ref24] Chattopadhyay D. K.; Webster D. C. Thermal stability and flame retardancy of polyurethanes. Prog. Polym. Sci. 2009, 34, 1068–1133. 10.1016/j.progpolymsci.2009.06.002.

[ref25] Wan L.; Deng C.; Chen H.; Zhao Z.-Y.; Huang S.-C.; Wei W.-C.; Yang A.-H.; Zhao H.-B.; Wang Y.-Z. Flame-retarded thermoplastic polyurethane elastomer: From organic materials to nanocomposites and new prospects. Chem. Eng. J. 2021, 417, 129314 10.1016/j.cej.2021.129314.

[ref26] Hobbs C. E. Recent advances in bio-based flame retardant additives for synthetic polymeric materials. Polymers 2019, 11, 224 10.3390/polym11020224. 30960208 PMC6419264

[ref27] Costes L.; Laoutid F.; Brohez S.; Dubois P. Bio-based flame retardants: When nature meets fire protection. Mater. Sci. Eng., R 2017, 117, 1–25. 10.1016/j.mser.2017.04.001.

[ref28] Cai W.; Wang B.; Liu L.; Zhou X.; Chu F.; Zhan J.; Hu Y.; Kan Y.; Wang X. An operable platform towards functionalization of chemically inert boron nitride nanosheets for flame retardancy and toxic gas suppression of thermoplastic polyurethane. Composites, Part B 2019, 178, 107462 10.1016/j.compositesb.2019.107462.

[ref29] Wang J.; Zhang D.; Zhang Y.; Cai W.; Yao C.; Hu Y.; Hu W. Construction of multifunctional boron nitride nanosheet towards reducing toxic volatiles (CO and HCN) generation and fire hazard of thermoplastic polyurethane. J. Hazard. Mater. 2019, 362, 482–494. 10.1016/j.jhazmat.2018.09.009. 30296673

[ref30] Cheng L.; Wang J.; Qiu S.; Wang J.; Zhou Y.; Han L.; Zou B.; Xu Z.; Hu Y.; Ma C. Supramolecular wrapped sandwich like SW-Si3N4 hybrid sheets as advanced filler toward reducing fire risks and enhancing thermal conductivity of thermoplastic polyurethanes. J. Colloid Interface Sci. 2021, 603, 844–855. 10.1016/j.jcis.2021.06.153. 34237602

[ref31] Kremer C.; Torres J.; Bianchi A.; Savastano M.; Bazzicalupi C. Myo-inositol hexakisphosphate: Coordinative versatility of a natural product. Coord. Chem. Rev. 2020, 419, 213403 10.1016/j.ccr.2020.213403.

[ref32] Crea F.; De Stefano C.; Milea D.; Sammartano S. Formation and stability of phytate complexes in solution. Coor. Chem. Rev. 2008, 252, 1108–1120. 10.1016/j.ccr.2007.09.008.

[ref33] Morgan A. B. Non-Halogenated Flame Retardant Handbook; John Wiley & Sons, 2021.

[ref34] Zheng Z.; Yang S. L. B. W. T.; Cui X.; Wang H. Preparation of a novel phosphorus-and nitrogen-containing flame retardant and its synergistic effect in the intumescent flame-retarding polypropylene system. Polym. Compos. 2015, 36, 1606–1619. 10.1002/pc.23069.

[ref35] Zheng Z.; Liu Y.; Dai B.; Meng C.; Guo Z. Synergistic effect of organically modified zinc aluminum layered double hydroxide in intumescent flame-retarding polypropylene composites containing melamine phytate and dipentaerythritol. Polym. Eng. Sci. 2019, 59, 2301–2312. 10.1002/pen.25233.

[ref36] Zhan Y.; Yuan B.; Shang S. Synergistic effect of layered melamine-phytate and intumescent flame retardant on enhancing fire safety of polypropylene. J. Therm. Anal. Calorim. 2022, 147, 285–295. 10.1007/s10973-020-10228-6.

[ref37] Shang S.; Yuan B.; Sun Y.; Chen G.; Huang C.; Yu B.; He S.; Dai H.; Chen X. Facile preparation of layered melamine-phytate flame retardant via supramolecular self-assembly technology. J. Colloid Interface Sci. 2019, 553, 364–371. 10.1016/j.jcis.2019.06.015. 31220710

[ref38] Li W.-X.; Zhang H.-J.; Hu X.-P.; Yang W.-X.; Cheng Z.; Xie C.-Q. Highly efficient replacement of traditional intumescent flame retardants in polypropylene by manganese ions doped melamine phytate nanosheets. J. Hazard. Mater. 2020, 398, 123001 10.1016/j.jhazmat.2020.123001. 32768832

[ref39] Wang D.; Wang Y.; Zhang X.; Li T.; Du M.; Chen M.; Dong W. Preferred zinc-modified melamine phytate for the flame retardant polylactide with limited smoke release. New J. Chem. 2021, 45, 13329–13339. 10.1039/D1NJ02219A.

[ref40] Yuan X.; Luo K.; Zhang K.; He J.; Zhao Y.; Yu D. Combinatorial vibration-mode assignment for the FTIR spectrum of crystalline melamine: A strategic approach toward theoretical IR vibrational calculations of triazine-based compounds. J. Phys. Chem. A 2016, 120, 7427–7433. 10.1021/acs.jpca.6b06015. 27598419

[ref41] Ma D.; Zhao P.; Li J. Effects of zinc phytate on flame retardancy and thermal degradation behaviors of intumescent flame-retardant polypropylene. Polym.-Plast. Technol. Eng. 2017, 56, 1167–1176. 10.1080/03602559.2016.1255754.

[ref42] Sakai H.; Ikemoto Y.; Kinoshita T.; Moriwaki T.; Yoshida K. T. Fourier-transform spectra of metal salts of phytic acid in the mid-to far-infrared spectral range. Vib. Spectrosc. 2017, 92, 215–219. 10.1016/j.vibspec.2017.07.003.

[ref43] Costa L.; Camino G.; Luda di Cortemiglia M. P. Mechanism of thermal degradation of fire-retardant melamine salts. 1990.

[ref44] Wang Z.; Lv P.; Hu Y.; Hu K. Thermal degradation study of intumescent flame retardants by TG and FTIR: Melamine phosphate and its mixture with pentaerythritol. J. Anal. Appl. Pyrolysis 2009, 86, 207–214. 10.1016/j.jaap.2009.06.007.

[ref45] Costa L.; Camino G. Thermal behaviour of melamine. J. Therm. Anal. 1988, 34, 423–429. 10.1007/BF01913181.

[ref46] Daneluti A. L. M.; do Rosário Matos J. Study of thermal behavior of phytic acid. Braz. J. Pharm. Sci. 2013, 49, 275–283. 10.1590/S1984-82502013000200009.

[ref47] Gao Y.-Y.; Deng C.; Du Y.-Y.; Huang S.-C.; Wang Y.-Z. A novel bio-based flame retardant for polypropylene from phytic acid. Polym. Degrad. Stab. 2019, 161, 298–308. 10.1016/j.polymdegradstab.2019.02.005.

[ref48] Senoz E.; Wool R. P.; McChalicher C. W.; Hong C. K. Physical and chemical changes in feather keratin during pyrolysis. Polym. Degrad. Stab. 2012, 97, 297–307. 10.1016/j.polymdegradstab.2011.12.018.

[ref49] Brebu M.; Spiridon I. Thermal degradation of keratin waste. J. Anal. Appl. Pyrolysis 2011, 91, 288–295. 10.1016/j.jaap.2011.03.003.

[ref50] Zhang X.; Zhou X.-Y.; Cheng X.-W.; Tang R.-C. Phytic acid as an eco-friendly flame retardant for silk/wool blend: A comparative study with fluorotitanate and fluorozirconate. J. Cleaner Prod. 2018, 198, 1044–1052. 10.1016/j.jclepro.2018.07.103.

[ref51] Hu P.; Zheng X.; Zhu J.; Wu B. Effects of chicken feather keratin on smoke suppression characteristics and flame retardancy of epoxy resin. Polym. Adv. Technol. 2020, 31, 2480–2491. 10.1002/pat.4963.

[ref52] Hampton C.; Demoin D.; Glaser R. E. Vibrational Spectroscopy Tutorial: Sulfur and Phosphorus; University of Missouri, 2010.

[ref53] Realinho V.; Haurie L.; Formosa J.; Velasco J. I. Flame retardancy effect of combined ammonium polyphosphate and aluminium diethyl phosphinate in acrylonitrile-butadiene-styrene. Polym. Degrad. Stab. 2018, 155, 208–219. 10.1016/j.polymdegradstab.2018.07.022.

[ref54] Xu W.; Cheng C.; Qin Z.; Zhong D.; Cheng Z.; Zhang Q. Improvement of thermoplastic polyurethane’s flame retardancy and thermal conductivity by leaf-shaped cobalt-zeolitic imidazolate framework–modified graphene and intumescent flame retardant. Polym. Adv. Technol. 2021, 32, 228–240. 10.1002/pat.5078.

